# Most consumed foods in Brazil: evolution between 2008-2009 and 2017-2018

**DOI:** 10.11606/s1518-8787.2021055003406

**Published:** 2021-11-16

**Authors:** Renata Muniz Rodrigues, Amanda de Moura Souza, Ilana Nogueira Bezerra, Rosangela Alves Pereira, Edna Massae Yokoo, Rosely Sichieri

**Affiliations:** I Universidade do Estado do Rio de Janeiro Instituto de Medicina Social Rio de Janeiro RJ Brasil Universidade do Estado do Rio de Janeiro. Instituto de Medicina Social. Rio de Janeiro, RJ, Brasil; II Universidade Federal do Rio de Janeiro Instituto de Estudos em Saúde Coletiva Rio de Janeiro RJ Brasil Universidade Federal do Rio de Janeiro. Instituto de Estudos em Saúde Coletiva. Rio de Janeiro, RJ, Brasil; III Universidade Estadual do Ceará Centro de Ciências da Saúde Fortaleza CE Brasil Universidade Estadual do Ceará. Centro de Ciências da Saúde. Fortaleza, CE, Brasil; IV Universidade Federal do Rio de Janeiro Departamento de Nutrição Social e Aplicada Rio de Janeiro RJ Brasil Universidade Federal do Rio de Janeiro. Departamento de Nutrição Social e Aplicada. Rio de Janeiro, RJ, Brasil; V Universidade Federal Fluminense Departamento de Epidemiologia e Bioestatística Niterói RJ Brasil Universidade Federal Fluminense. Departamento de Epidemiologia e Bioestatística. Niterói, RJ, Brasil

**Keywords:** Food Consumption, Feeding Behavior, Staple Food, Diet Surveys

## Abstract

**OBJECTIVE:**

To describe the evolution of food consumption by the Brazilian population in 2008–2009 to 2017–2018.

**METHODS:**

Data from the National Dietary Surveys of 2008–2009 and 2017–2018 were used. Both surveys estimated food consumption of two non-consecutive days of individuals aged 10 years or older. The first survey collected consumption data from 34,003 individuals through food records; the second, obtained data from 46,164 individuals, through 24-hour recalls. The twenty most frequently reported food groups in the two surveys were identified. The probability of consumption of each food group in the two surveys was estimated according to sex, age and income. This study presents the foods that had a change in the frequency of consumption of 5% or higher between the two surveys. The probability of consumption was corrected for intra-individual variability using the method developed by the National Cancer Institute.

**RESULTS:**

Rice, beans, coffee, bread, vegetables and beef remained the staple Brazilian diet, ranking as the six most consumed items in both surveys. Ultra-processed foods such as sweet/stuffed cookies, savory cookies, processed meats and carbonated drinks also remained among the 20 most consumed foods. Trend analyses showed, regardless of gender, age and income range, a decrease in the consumption of rice, beans, beef, bread, fruit, milk and dairy, processed meats and carbonated drinks, and an increase in the consumption of sandwiches.

**CONCLUSION:**

The Brazilian diet is still characterized by consumption of traditional foods, such as rice and beans, and by high frequency of consumption of ultra-processed foods, such as cookies and carbonated drinks. However, between the years of 2008–2009 and 20172018, there was a decrease in the consumption of rice, beans, beef, bread, fruit, milk and dairy, processed meats and carbonated drinks, but an increase in the consumption of sandwiches. The results show a decrease in quality in the Brazilian diet.

## INTRODUCTION

Changes in the economy, in food and nutrition policies, and in food processing can affect the population's eating pattern^1^. Data from the Family Budget Survey ( *Pesquisa de Orçamentos Familiares – POF* ), between the periods 2002–2003 and 2017–2018, showed a decline in the calorie share of fresh food and an increase in the share of ultra-processed foods^2^, the consumption of which has been associated with increased risk of obesity and other chronic non-communicable diseases (NCDs)^3 , 4^. At the same time, in the period between 2013 and 2018, which saw the Brazilian economy stagnate, the proportion of food secure households fell from 77% to 63%^5^.

Assessing changes in the population's food consumption pattern is essential for finding challenges and opportunities for health interventions, informing policy makers and support public nutrition policies^1^. For many years, household food availability data were the only ones available to identify changes in the population's food consumption patterns. In the last two POF, a module entitled National Dietary Survey ( *Inquérito Nacional de Alimentação – INA* ) was included to estimate individual food consumption, prevalence of food group intake and nutritional inadequacies, according to sex and age group.

The objective of this study is to describe the evolution of food consumption of the Brazilian population using data from the INA of 2008–2009 and 2017–2018.

## METHODS

The study used data of the INA included in the POFs of 2008–2009 and 2017–2018. Details about the sampling and data collection were published by the Brazilian Institute of Geography and Statistics ^a^ .

In summary, the sample design adopted in the two POFs was the same, based on a master sample composed of a set of census sectors. These sectors were stratified considering the administrative division of the location of the household, the geographical area, the situation (urban or rural) and the income of the family head based on the 2000 Census for the POF of 2008–2009 and in the 2010 Census for the POF of 2017–2018. In each POF, a subsample of the sectors of the master sample was selected by simple random sampling to compose the consumption sample of the INA. All residents of the household older than 10 years participated.

All participants were instructed to provide information on their individual food consumption. In the POF of 2008–2009, 4,696 sectors and 55,970 households participated in the interviews. Of these, 13,569 presented food consumption data for 34,003 individuals. In the POF of 2017–2018, 5,504 sectors and 57,920 households participated in the interviews. Of these, 20,112 presented food consumption data for a total of 46,164 individuals. In both surveys, the sample was able to represent the urban and rural populations, the different Brazilian regions and socioeconomic levels.

In 2008–2009, consumption data were collected using two non-consecutive food records, in which respondents recorded all the food and beverages consumed throughout the day, including information about the time and place of consumption, quantity and, in the case of certain foods, such as meats and vegetables, the cooking method. The records were reviewed, corrected and stored in a computer program developed specifically for this purpose. The research agents performed these steps with the respondents at their homes.

In 2017–In 2018, consumption data were obtained using two 24-hour dietary recalls, completed on non-consecutive days in a personal interviews conducted by a research agent trained in the multiple-pass method^6^. The interviewees provided detailed information: unit of measurement and quantity, times and places of consumption, meal occasions and form of cooking for specific foods. Probing questions were asked about the addition of olive oil, butter, margarine, sugar, sweetener, honey, molasses, mayonnaise, ketchup, mustard, soy sauce, grated cheese and cream to the reported foods.

INA participants listed 1,121 food items in 2008–2009, and 1,593 items in 2017–2018. In both surveys, the food items were classified into 79 groups. This study estimates the frequency of reporting consumption of these groups based on the first day of the food survey, excluding from the comparative analysis the groups of oils and fats, sweeteners and honey/molasses and sugar, since the collection instrument specified additions of these products only in the second survey.

Among the 20 most frequently reported food groups, a trend analysis of the relative frequency of consumption was performed for groups exhibiting change of 5% or more in the frequency of consumption between the two surveys. They were rice, beans, beef, bread, fruit, milk and dairy, carbonated drinks, processed meats and sandwiches.

Frequency rates were estimated according to sex, age group (adolescents: ages 10 to 19; adults: ages 20 to 59; and elderly: from age 60) and income group (up to ½ minimum salary, between ½ and one minimum salary, between one and two minimum salary and more than two minimum salaries). Additionally, the average consumption (in grams per day) is presented for the foods and food groups selected according to income ranges.

The probability of consumption of each of the food groups in the two surveys was estimated using a method developed by National Cancer Institute (NCI), which uses a two-part model to estimate episodic food consumption^7 , 8^. The first part of the model estimates the probability of consumption using logistic regression, and the second estimates the amount consumed using linear regression. The two parts are combined to produce an estimate of the usual consumption of each food group^8^. This analysis used data from the two days of food consumption.

The odds ratio (OR) of intake of each food group and its respective 95% confidence interval (95%CI) were estimated considering the probability of consumption of the food groups as a dependent variable and the year of the survey as an independent variable. The reference survey was the INA of 2008–2009. The models were developed according to sex, age groups and income groups, using the macros mixtran and distribution from the NCI ^b^ . All analyses were adjusted by age and considered the complexity of the sample design and the expansion factor of the research. The Statistical Analysis System version 9.4 was used.

## RESULTS

**Figure 1:**
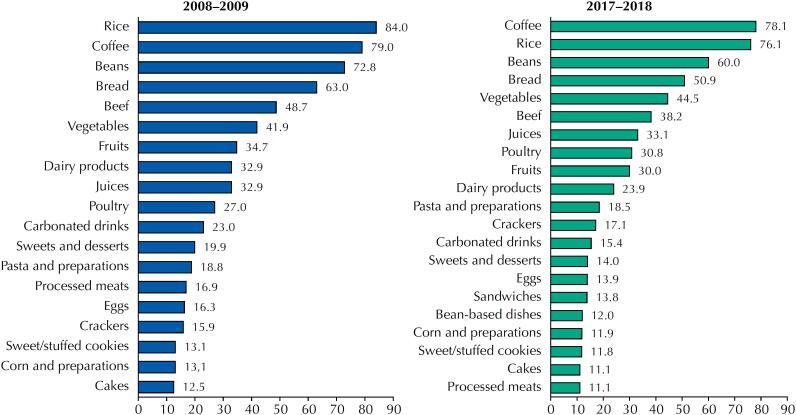
Foods with higher frequency of consumption, INA 2008–2009 and 2017–2019, Brazil.

Rice, beans, coffee, bread, vegetables and beef remained as the staple items of the Brazilian diet. These food groups were among the six most consumed ones in both surveys ( Figure 1 ). In the INA of 2017–2018, the food groups ranking among the 20 most consumed, and which were not in the first survey, were sandwiches and bean-based dishes ( Figure 1 ). Conversely, ultra-processed foods, such as sweet/stuffed cookies, crackers and carbonated drinks, remained among the 20 most consumed.

Trend analyses showed a decrease in the consumption of rice, beans, beef, bread, fruit, milk and dairy, processed meats and carbonated drinks, as well as an increase in the consumption of sandwiches ( Table 1 ). These changes occurred regardless of sex and age. The decrease was statistically significant for all food groups analyzed, except for the fruit group among elderly men (CR = 0.75; 95%CI: 0.47–1.20) and carbonated drinks among elderly women (CR = 0.55; 95%CI: 0.24–1.26).

**Table 1:** Adjusted odds ratio b and 95% confidence interval of consumption of food groups by sex and age group, National Food Surveys 2008–2009 and 2017–2018.

Food Groups	Male Sex			Female Sex		
	Adolescent	Adult	Elderly	Adolescent	Adult	Elderly
	OR (IC95%)	OR (IC95%)	OR (IC95%)	OR (IC95%)	OR (IC95%)	OR (IC95%)
Rice	0.53 (0.43–0.66)	0.38 (0.33–0.45)	0.35 (0.29–0.43)	0.62 (0.44–0.87)	0.47 (0.42–0.53)	0.30 (0.23–0.38)
Beans	0.30 (0.26–0.35)	0.32 (0.28–0.36)	0.29 (0.21–0.40)	0.42 (0.33–0.53)	0.41 (0.36–0.47)	0.36 (0.22–0.61)
Beef	0.68 (0.57–0.81)	0.62 (0.58–0.67)	0.66 (0.60–0.73)	0.62 (0.57–0.68)	0.68 (0.61–0.76)	0.64 (0.52–0.79)
Fruit	0.41 (0.23–0.73)	0.52 (0.48–0.59)	0.75 (0.47–1.20)	0.50 (0.44–0.56)	0.63 (0.53–0.76)	0.72 (0.56–0.94)
Dairy products	0.48 (0.34–0.67)	0.39 (0.33–0.46)	0.54 (0.36–0.83)	0.44 (0.29–0.66)	0.41 (0.36–0.46)	0.43 (0.35–0.54)
Bread	0.48 (0.40–0.59)	0.41 (0.34–0.49)	0.54 (0.34–0.88)	0.40 (0.32–0.51)	0.39 (0.35–0.43)	0.38 (0.31–0.46)
Processed meats	0.63 (0.54–0.74)	0.53 (0.49–0.58)	0.51 (0.34–0.75)	0.70 (0.65–0.76)	0.55 (0.49–0.63)	0.61 (0.39–0.96)
Carbonated drinks	0.67 (0.61–0.75)	0.54 (0.46–0.64)	0.56 (0.41–0.78)	0.52 (0.39–0.71)	0.54 (0.49–0.60)	0.55 (0.24–1.26)
Sandwiches	2.58 (2.11–3.16)	3.01 (2.27–4.00)	4.02 (1.90–8.51)	1.76 (1.52–2.03)	3.01 (2.27–4.00)	4.96 (3.05–8.05)

^a^ Age-adjusted.

b Reference year: 2008–2009.

In both sexes, the decrease in rice consumption was more pronounced in the elderly (OR = 0.35; 95%CI: 0.29­–0.43 among men *versus* OR = 0.30; 95%CI: 0.23–0.38 among women). The two biggest changes, considering all age groups and both genders, were decreased consumption of beans and increased consumption of sandwiches, which showed an estimate of more than 4 times more chances among the elderly (OR = 4.96; 95%CI: 3.05–8.05).

Similar results were observed in all income groups, with decreased consumption of rice, beans, beef, bread, fruit, milk and dairy, processed meats and carbonated drinks, and increased consumption of sandwiches. The trend observed was statistically significant for all food groups in all categories analyzed, with the exception of processed meats (OR = 1.08; 95%CI: 0.88–1.34) and carbonated drinks (OR = 0.90; 95%CI: 0.73–1.11) in the lowest income category ( Table 2 ). The decrease in the consumption of rice, bread, processed meats and carbonated drinks was more pronounced in the higher income category when compared to the lower income category. Conversely, the trend of increased consumption of sandwiches was higher in the lower income category (OR = 3.54; 95%CI: 2.83–4.42).

**Table 2:** Adjusted odds ratio b and 95% confidence interval of consumption of food groups according to categories of minimum sarlaries per capita, National Food Surveys 2008–2009 and 2017–2018.

Food groups	Minimum salaries (MS)			
	< 0.5 MS	0.5 to 1 MS	1 to 2 MS	> 2 MS
	OR (IC95%)	OR (IC95%)	OR (IC95%)	OR (IC95%)
Rice	0.61 (0.40–0.94)	0.67 (0.58–0.77)	0.31 (0.23 –0.41)	0.35 (0.30–0.40)
Beans	0.24 (0.13–0.43)	0.39 (0.32–0.48)	0.29 (0.26–0.32)	0.42 (0.35–0.50)
Beef	0.58 (0.43–0.78)	0.55 (0.51–0.61)	0.67 (0.64–0.70)	0.71 (0.63–0.80)
Fruit	0.44 (0.27–0.69)	0.58 (0.49–0.67)	0.65 (0.52–0.82)	0.68 (0.64–0.72)
Dairy products	0.40 (0.29–0.55)	0.38 (0.31–0.48)	0.51 (0.48–0.54)	0.46 (0.41–0.52)
Bread	0.82 (0.68–0.98)	0.45 (0.39–0.53)	0.40 (0.35–0.45)	0.26 (0.23–0.29)
Processed meats	1.08 (0.88–1.34)	0.63 (0.52–0.76)	0.56 (0.51–0.63)	0.39 (0.35–0.43)
Carbonated drinks	0.90 (0.73–1.11)	0.62 (0.55–0.69)	0.68 (0.58–0.78)	0.40 (0.31–0.51)
Sandwiches	3.54 (2.83–4.42)	3.09 (2.42–3.94)	2.81 (2.20–3.58)	2.75 (2.27–3.32)

MS: minimum salary.

^a^ Adjusted by age and gender.

b Reference year: 2008–2009.

**Figure 2:**
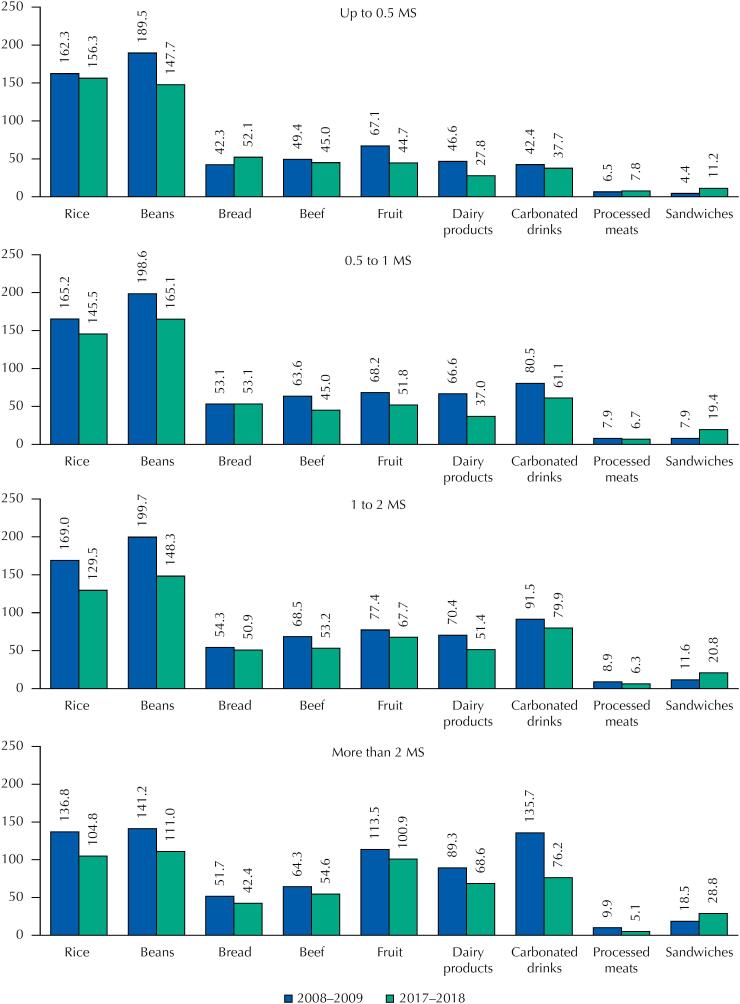
Average food consumption (g/day) by minimum salary categories (MS) per capita, INA 2008–2009 and 2017–2018, Brazil.

Figure 2 shows the average consumption, in grams per day, of food groups according to income ranges. The results show similarities with the consumption frequencies in general: decreased consumption of rice, beans, beef, bread, fruit, milk and dairy, processed meats and carbonated drinks, and increased consumption of sandwiches. For the first category of income (up to ½ minimum salary per capita), there was a decrease between the surveys in the average consumption of beans, fruit and milk and dairy 41.8 g/day, 22.4 g/day and 18.8 g/day, respectively. However, in this same income category, there was an increase in the average consumption of bread, from 42.3 g in 2008–2009 to 52.1 g in 2017–2018. In the higher income category (more than two minimum salaries per capita), there was a significant decrease in the average consumption of carbonated drinks (from 135.7 g/day to 76.2 g/day) and processed meats (from 9.9 g/day to 5.1 g/day), while the average consumption of sandwiches in this same income category increased by 10g in the period (18.5 g/day to 28.8 g/day).

## DISCUSSION

This detailed study on the evolution of food consumption in the last 10 years shows that the Brazilian diet is still characterized by consumption of traditional foods, such as rice and beans, and high frequency of ultra-processed foods consumption, such as cookies and carbonated drinks. However, in the period from 2008–2009 to 2017–2018, there was a decrease in the frequency of consumption of rice, beans, fruit, beef, bread, milk and dairy, processed meats and carbonated drinks, but an increase in the consumption of sandwiches. This trend was observed regardless of sex, age and income range.

Although the traditional food items of the Brazilian diet remain the most consumed, the decrease in the consumption of these foods between the two surveys draws attention, especially the beans group, as it is an important healthy diet marker in the country. Beans are a spontaneously available food item among the population and an important part of Brazil's food culture. It is known that dietary patterns based on traditional foods of the Brazilian diet, fresh and minimally processed, are related to lower occurrence of obesity and less risk of NCDs^9^.

Our findings corroborate the results of other studies with national representativeness. POF data showed that the average per capita annual household availability of rice and beans fell by 37% in 2002–2003 and by 52% in 2017–2018^2^. Data from the Surveillance System of Risk Factors for Chronic Non-Communicable Diseases (Vigitel) also showed a decrease in the regular consumption of beans between 2006 and 2019: an average annual variation of -0.58 percentage point among men and -0.57 among women^10^.

On the other hand, the decrease in the consumption of processed meats and carbonated drinks was a positive aspect, considering that these are highly processed, nutritionally unbalanced food items that lead to excessive energy consumption^11^. This reduction was also observed by the Vigitel between 2006 and 2019, with an average annual variation in the frequency of consumption of carbonated drinks of -1.52 percentage point among men and -1.30 among women^10^.

The reduction in the consumption of both traditional and ultra-processed foods may have been driven by the economic crisis that began in 2015^12^, which increased food insecurity in the country. According to data of the 2017–2018 POF, food insecurity increased from 22.6% in 2013 to 36.7% in 2018, and severe food insecurity increased by 43.7% in that period^5^.

The increased frequency of consumption of sandwiches also needs to be discussed, as it reflects an important change in dietary habits in the substitution of traditional meals with snacks and convenience foods. Sandwiches are often prepared with ultra-processed products, which have high calorie content and excess sodium or sugar, which can have a negative health impact^13^.

The changes observed in food consumption were similar among the age groups, but the decrease in fruit consumption was more pronounced among adolescents. Generational differences in the consumption of fruit and other healthy foods have been observed previously^14^. Seniors and older adults are more likely to make positive dietary changes due to greater understanding of the benefits of a proper diet, or due to chronic diseases requiring the adoption of healthier habits^15^.

The assessment of consumption according to age groups shows that the diet of adolescents already had negative characteristics compared to that of elders in the 2008–2009 survey. Ten years later, the diet of adolescents remains of lower quality than that of elders, which is consistent with the increased occurrence of overweight and obesity among Brazilian adolescents in recent years^16^. Overweight in adolescence brings important losses throughout life, as the behavioral and lifestyle characteristics acquired at this phase tend to continue into adulthood^16^.

Another worrisome fact to be highlighted is the sharpest increase in the frequency of consumption of sandwiches among the elderly, in view of the arguments presented earlier (replacement of meals with snacks and use of ultra-processed products as ingredients). Consumption of these foods was already higher among adolescents in the first survey. Therefore, the increased frequency of sandwich consumption among this group was lower compared to the high increase among adults and the elders.

The 10-year period between the analyzed surveys also includes changes in the age composition of the population, which may have influenced findings related to changes in dietary habits. The life expectancy of Brazilians increased more than three years in this period, reflecting an improvement in quality of life, which enables less limiting behaviors among elders^17^. This was also observed in the increased consumption and calorie contribution of food outside the home among elders, reinforcing the hypothesis that greater socialization brings them closer to behaviors of younger adults^18^. In this study, for the analysis of trends, the models were adjusted by age to minimize the effect of this variable on the changes in dietary patterns. Furthermore, the effects of changes, stratified by age group, were presented.

The variation in the consumption of the food groups evaluated was not uniform throughout the different income groups. The decreased consumption of rice, bread, processed meats and carbonated drinks was more pronounced in the higher income group. However, an inverse trend without statistical significance was observed for beans, beef and fruit, with the sharpest decrease occurring in the lower income range.

Similar results were observed in household food availability data in the period between 2008–2009 and 2017–2018 for the highest fifth of income: while the calorie share of rice decreased (from 12.3% to 10.9%), the calorie contribution of fruit increased (from 3.1% to 3.9%)^2^. These data confirm the disparities in habits between socioeconomic levels, showing the importance of policies aimed at improving living conditions, such as the Disability Allowance and the *Programa Bolsa Família* , which increase access to healthy food^19^. Strategies to limit access to unhealthy foods, such as carbonated drinks, were also discussed^20 , 21^.

Some limitations of this study need to be considered. Changes in the database, such as the addition of several items in the second survey, may have influenced the findings – for instance, the increased consumption of sandwiches observed. In the INA of 2008–2009, there were 17 codes available to record sandwich consumption, while in 2017–2018 this number increased to 61. Another possible limitation concerns the different methods applied to collect food consumption information: in 2008–2009, the food record was used, while in 2017–2018 the 24-hour food recall was used. However, Rodrigues et al. showed that it is possible to analyze the evolution of food consumption regardless of the method used in each survey by using analysis strategies for harmonization^22^.

A strong point of the study is the estimation of the usual food consumption of individuals. The two days’ worth of consumption collected in the two surveys allowed the estimation of intra-individual variability, incorporating the NCI method into the trend analyses, which allows estimating the distribution and effects of non-dietary co-variates on usual food consumption.

The study, which investigated actual food consumption of individuals aged 10 and older, is the first to assess food consumption trends in a representative sample of the Brazilian population based on individual data on global food consumption. Due to the detailed information on cooking methods and additions specific to foods consumed individually, the analyses presented overcome the bias of trends estimated with data on food availability.

The changes in the last decade, characterized by a decrease in the consumption of traditional foods, such as rice, beans and meat, and an increase in the consumption of sandwiches, signal a decrease in the quality of the Brazilian diet. Despite the decrease in the consumption of some ultra-processed foods, such as carbonated drinks and cookies, these items are still among the most consumed foods in the country. These results indicate that economic and public health measures are needed to allow and stimulate the consumption of beans, fruit and vegetables (important markers of dietary quality), while discouraging the consumption of ultra-processed foods. These measures would increase food and nutrition security and prevent NCDs, including obesity.

## Footnotes

a Instituto Brasileiro de Geografia e Estatística, Diretoria de Pesquisas, Coordenação de Trabalho e Rendimento. Pesquisa de Orçamentos Familiares, 2017–2018: análise do consumo alimentar pessoal. Rio de Janeiro: IBGE; 2020 [cited 2020 Oct 28]. Available from: https://biblioteca.ibge.gov.br/visualizacao/livros/liv101742.pdf

b National Institutes of Health (US), National Cancer Institute, Division of Cancer Control & Population Sciences. Epidemiology and Genomics Research Program. Usual dietary intakes: SAS macros for analysis of a single dietary compoment. Bethesda, MD: NCI; 2020 [cited 2020 Sep 14]. Available from: https://epi.grants.cancer.gov/diet/usualintakes/macros_single.html
