# Supplementary material for: Most consumed foods in Brazil: evolution between 2008-2009 and 2017-2018
Source: Rev Saude Publica. 2021 Nov 16;55(Supl 1):4s. doi: 10.11606/s1518-8787.2021055003406 (PMC8659613; doi:10.11606/s1518-8787.2021055003406)
Supplement: Translation

# Translation: Evolução dos alimentos mais consumidos no Brasil entre 2008-2009 e 2017-2018

**Authors:** Renata Muniz Rodrigues, Amanda de Moura Souza, Ilana Nogueira Bezerra, Rosangela Alves Pereira, Edna Massae Yokoo, Rosely Sichieri

**Affiliations:** I Rio de Janeiro RJ Brasil Universidade do Estado do Rio de Janeiro. Instituto de Medicina Social. Rio de Janeiro, RJ, Brasil; II Rio de Janeiro RJ Brasil Universidade Federal do Rio de Janeiro. Instituto de Estudos em Saúde Coletiva. Rio de Janeiro, RJ, Brasil; III Fortaleza CE Brasil Universidade Estadual do Ceará. Centro de Ciências da Saúde. Fortaleza, CE, Brasil; IV Rio de Janeiro RJ Brasil Universidade Federal do Rio de Janeiro. Departamento de Nutrição Social e Aplicada. Rio de Janeiro, RJ, Brasil; V Niterói RJ Brasil Universidade Federal Fluminense. Departamento de Epidemiologia e Bioestatística. Niterói, RJ, Brasil

**Keywords:** Consumo de Alimentos, Comportamento Alimentar, Alimentação Básica, Inquéritos sobre Dietas

## Abstract

**OBJETIVO:**

Descrever a evolução do consumo alimentar da população brasileira de 2008–2009 a 2017–2018.

**MÉTODOS:**

Foram utilizados dados dos Inquéritos Nacionais de Alimentação de 2008–2009 e 2017–2018, que estimaram o consumo alimentar de dois dias não consecutivos de indivíduos com 10 anos ou mais de idade. O primeiro inquérito colheu dados de consumo de 34.003 indivíduos por meio de registro alimentar; o segundo, de 46.164 indivíduos, por meio de recordatório de 24 horas. Identificaram-se os 20 grupos de alimentos mais frequentemente referidos nos dois inquéritos. A probabilidade de consumo de cada um dos grupos de alimentos nos dois inquéritos foi estimada segundo sexo, idade e renda. No presente estudo, são apresentados os alimentos que tiveram mudança igual ou superior a 5% na frequência de consumo entre os dois inquéritos. A probabilidade de consumo foi corrigida para a variabilidade intraindividual, utilizando método desenvolvido pelo National Cancer Institute.

**RESULTADOS:**

Arroz, feijão, café, pães, hortaliças e carne bovina permaneceram como base da alimentação dos brasileiros, sendo os seis itens mais consumidos em ambos os inquéritos. Alimentos ultraprocessados, como biscoitos doces/recheados, biscoitos salgados, carnes processadas e refrigerantes, também se mantiveram entre os 20 alimentos mais consumidos. As análises de tendência evidenciaram, independentemente de sexo, idade e faixa de renda, a diminuição do consumo de arroz, feijão, carne bovina, pães, frutas, laticínios, carnes processadas e refrigerantes, e o aumento da ingestão de sanduíches.

**CONCLUSÃO:**

A dieta do brasileiro permanece caracterizada pelo consumo de alimentos tradicionais, como arroz e feijão, e pela frequência elevada de ingestão de alimentos ultraprocessados, como biscoitos e refrigerantes. No entanto, entre os anos de 2008–2009 e 2017–2018, observou-se redução no consumo de arroz, feijão, carne bovina, pães, frutas, laticínios, carnes processadas e refrigerantes, mas aumento no consumo de sanduíches. Os resultados sinalizam piora na qualidade da alimentação do brasileiro.

## INTRODUÇÃO

Mudanças na economia, nas políticas de alimentação e nutrição e no processamento de alimentos podem afetar o padrão de consumo alimentar da população^1^. Segundo dados da Pesquisa de Orçamentos Familiares (POF), entre os períodos 2002–2003 e 2017–2018, observou-se declínio na participação calórica de alimentos *in natura* e aumento na participação de alimentos ultraprocessados^2^, cujo consumo tem sido associado a risco aumentado de obesidade e outras doenças crônicas não transmissíveis (DCNT)^3 , 4^. Paralelamente, no período entre 2013 e 2018, marcado pela estagnação da economia brasileira, a proporção de domicílios em segurança alimentar caiu de 77% para 63%^5^.

Avaliar mudanças no padrão de consumo alimentar da população é essencial para identificar desafios e oportunidades para intervenções em saúde, assim como para informar gestores e subsidiar políticas públicas em nutrição^1^. Por muitos anos, os dados de disponibilidade domiciliar foram os únicos à disposição para identificar mudanças nos padrões de consumo alimentar da população. Nas duas últimas POF, um módulo intitulado Inquérito Nacional de Alimentação (INA) foi incluído para estimar o consumo alimentar individual, a prevalência de ingestão de grupos de alimentos e inadequações nutricionais, segundo sexo e faixa etária.

O objetivo do presente estudo é descrever a evolução do consumo alimentar da população brasileira utilizando dados dos INA de 2008–2009 e 2017–2018.

## MÉTODOS

Foram utilizados os dados dos INA incluídos nas POF de 2008–2009 e 2017–2018. Detalhes sobre a amostragem e coleta de dados foram publicados pelo Instituto Brasileiro de Geografia e Estatística ^a^ .

Em síntese, o plano amostral adotado nas duas POF foi o mesmo, baseado em uma amostra mestra composta por um conjunto de setores censitários. Esses setores foram estratificados considerando a divisão administrativa da localização do domicílio, a área geográfica, a situação (urbana ou rural) e a renda do responsável familiar com base no Censo de 2000 para a POF 2008–2009 e no Censo de 2010 para a POF 2017–2018. Em cada POF, uma subamostra dos setores da amostra mestra foi selecionada por amostragem aleatória simples para compor a amostra de consumo do INA, com participação de todos os moradores da residência com mais de 10 anos de idade.

Todos os participantes foram orientados a fornecer informações sobre consumo alimentar individual. Na POF 2008–2009, 4.696 setores e 55.970 domicílios participaram das entrevistas. Destes, 13.569 apresentaram dados de consumo alimentar para 34.003 indivíduos. Na POF 2017–2018, 5.504 setores e 57.920 domicílios participaram das entrevistas. Destes, 20.112 apresentaram dados de consumo alimentar para um total de 46.164 indivíduos. Em ambas as pesquisas, a amostra foi capaz de representar a população urbana e rural, as diferentes regiões brasileiras e níveis socioeconômicos.

Em 2008–2009, os dados de consumo foram coletados por meio de dois registros alimentares não consecutivos, nos quais os respondentes anotavam todos os alimentos e bebidas consumidos ao longo do dia, incluindo informações sobre horário e local do consumo, quantidade e, no caso de determinados alimentos, como carnes e legumes, modo de preparação. Os registros foram revisados, corrigidos e armazenados em programa de computador desenvolvido especificamente para esse fim. Os agentes de pesquisa cumpriam essas etapas com o respondente, no próprio domicílio.

Em 2017–2018, os dados de consumo foram obtidos por meio de dois recordatórios alimentares de 24 horas, preenchidos em dias não consecutivos em entrevista pessoal com agente de pesquisa treinado no método de múltiplas passagens^6^. Os entrevistados forneciam informações detalhadas: unidade de medida e quantidade, horários e locais de consumo, ocasião das refeições e forma de preparo para alimentos específicos. Foram realizadas perguntas de sondagem sobre a adição de azeite, manteiga, margarina, açúcar, adoçante, mel, melado, maionese, ketchup, mostarda, molho shoyu, queijo ralado e creme de leite aos alimentos relatados.

Os participantes do INA citaram 1.121 itens alimentares em 2008–2009, e 1.593 itens em 2017–2018. Em ambos os inquéritos os itens alimentares foram classificados em 79 grupos. O presente estudo estima a frequência de relato do consumo desses grupos com base no primeiro dia de inquérito alimentar, excluindo das análises comparativas os grupos de óleos e gorduras, adoçantes e mel/rapadura e açúcar de mesa, pois somente no segundo inquérito o instrumento de coleta especificava adições desses produtos.

Entre os 20 grupos de alimentos mais frequentemente referidos, realizou-se análise de tendência da frequência relativa de consumo para os grupos que tiveram mudança igual ou superior a 5% na frequência de consumo entre os dois inquéritos. Foram eles: arroz, feijão, carne bovina, pães, frutas, laticínios, refrigerantes, carnes processadas e sanduíches.

As frequências foram estimadas segundo sexo, faixa etária (adolescentes: de 10 a 19 anos de idade; adultos: de 20 a 59 anos; e idosos: 60 anos ou mais) e faixa de renda (até ½ salário-mínimo, entre ½ e um salário-mínimo, entre um e dois salários-mínimos e mais de dois salários-mínimos). Adicionalmente, são apresentadas as médias de consumo (em gramas por dia) para os alimentos e grupos alimentares selecionados segundo as faixas de renda.

A probabilidade de consumo de cada um dos grupos alimentares nos dois inquéritos foi estimada com método desenvolvido pelo *National Cancer Institute* (NCI), que utiliza um modelo de duas partes para estimar o consumo episódico de alimentos^7 , 8^. A primeira parte do modelo estima a probabilidade de consumo usando regressão logística, e a segunda estima a quantidade consumida por meio de regressão linear. As duas partes são combinadas para se chegar a uma estimativa do consumo usual de cada grupo de alimentos^8^. Para esta análise foram utilizados os dados dos dois dias de consumo alimentar.

A razão de chances (RC) de ingestão de cada grupo de alimentos e seu respectivo intervalo de confiança de 95% (IC95%) foram estimados considerando a probabilidade de consumo dos grupos de alimentos como variável dependente e o ano de realização do inquérito como variável independente, sendo o INA 2008–2009 o inquérito de referência. Os modelos foram desenvolvidos segundo sexo, faixas etárias e faixas de renda, utilizando as macros *mixtran* e *distrib* do NCI ^b^ . Todas as análises foram ajustadas por idade e consideraram a complexidade do desenho amostral e o fator de expansão da pesquisa, utilizando o programa Statistical Analysis System versão 9.4.

## RESULTADOS

Arroz, feijão, café, pães, hortaliças e carne bovina permaneceram como os alimentos-base da alimentação do brasileiro. Esses grupos de alimentos figuram entre os seis mais consumidos em ambos os inquéritos ( Figura 1 ).

**Figura 1:** Alimentos com maior frequência de consumo, INA 2008–2009 e 2017–2019, Brasil

No INA 2017–2018, os grupos de alimentos que apareceram entre os 20 mais consumidos e que não estavam no primeiro inquérito foram os sanduíches e as preparações à base de feijão ( Figura 1 ). Por outro lado, alimentos ultraprocessados, como biscoitos doces/recheados, biscoitos salgados e refrigerantes, mantiveram-se entre os 20 mais consumidos.

As análises de tendência evidenciaram diminuição do consumo de arroz, feijão, carne bovina, pães, frutas, laticínios, carnes processadas e refrigerantes, e aumento da ingestão de sanduíches ( Tabela 1 ). Essas mudanças ocorreram independentemente de sexo e idade. A redução foi estatisticamente significante para todos os grupos de alimentos analisados, exceto para o grupo das frutas entre homens idosos (RC = 0,75; IC95% 0,47–1,20) e refrigerantes entre mulheres idosas (RC = 0,55; IC95% 0,24–1,26).

**Tabela 1:** Razão de chances ajustada a , b e intervalo de confiança de 95% de consumo de grupos alimentares segundo sexo e faixa etária, Inquéritos Nacionais de Alimentação 2008–2009 e 2017–2018.

Grupos Alimentares	Sexo Masculino			Sexo Feminino		
	Adolescentes	Adultos	Idosos	Adolescentes	Adultas	Idosas
	RC (IC95%)	RC (IC95%)	RC (IC95%)	RC (IC95%)	RC (IC95%)	RC (IC95%)
Arroz	0,53 (0,43–0,66)	0,38 (0,33–0,45)	0,35 (0,29–0,43)	0,62 (0,44–0,87)	0,47 (0,42–0,53)	0,30 (0,23–0,38)
Feijão	0,30 (0,26–0,35)	0,32 (0,28–0,36)	0,29 (0,21–0,40)	0,42 (0,33–0,53)	0,41 (0,36–0,47)	0,36 (0,22–0,61)
Carne bovina	0,68 (0,57–0,81)	0,62 (0,58–0,67)	0,66 (0,60–0,73)	0,62 (0,57–0,68)	0,68 (0,61–0,76)	0,64 (0,52–0,79)
Frutas	0,41 (0,23–0,73)	0,52 (0,48–0,59)	0,75 (0,47–1,20)	0,50 (0,44–0,56)	0,63 (0,53–0,76)	0,72 (0,56–0,94)
Laticínios	0,48 (0,34–0,67)	0,39 (0,33–0,46)	0,54 (0,36–0,83)	0,44 (0,29–0,66)	0,41 (0,36–0,46)	0,43 (0,35–0,54)
Pães	0,48 (0,40–0,59)	0,41 (0,34–0,49)	0,54 (0,34–0,88)	0,40 (0,32–0,51)	0,39 (0,35–0,43)	0,38 (0,31–0,46)
Carnes processadas	0,63 (0,54–0,74)	0,53 (0,49–0,58)	0,51 (0,34–0,75)	0,70 (0,65–0,76)	0,55 (0,49–0,63)	0,61 (0,39–0,96)
Refrigerantes	0,67 (0,61–0,75)	0,54 (0,46–0,64)	0,56 (0,41–0,78)	0,52 (0,39–0,71)	0,54 (0,49–0,60)	0,55 (0,24–1,26)
Sanduíches	2,58 (2,11–3,16)	3,01 (2,27–4,00)	4,02 (1,90–8,51)	1,76 (1,52–2,03)	3,01 (2,27–4,00)	4,96 (3,05–8,05)

a Ajustada por idade

b Ano de referência 2008–2009.

Em ambos os sexos, a redução do consumo de arroz foi mais acentuada em idosos (RC = 0,35; IC95% 0,29–0,43 entre os homens *versus* RC = 0,30; IC95% 0,23–0,38 entre as mulheres). As duas maiores mudanças, considerando todas as faixas etárias e ambos os sexos, foram a redução do consumo do feijão e aumento do consumo de sanduíches, que apresentou estimativa de mais de 4 vezes mais chances entre as idosas (RC = 4,96; IC95% 3,05–8,05).

Resultados semelhantes foram observados em todas as faixas de renda, com redução do consumo para os grupos do arroz, feijão, carne bovina, pães, frutas, laticínios, carnes processadas e refrigerantes, e elevação da ingestão de sanduíches. A tendência observada foi estatisticamente significante para todos os grupos de alimentos em todas as categorias analisadas, com exceção das carnes processadas (RC = 1,08; IC95% 0,88–1,34) e refrigerantes (RC = 0,90; IC95% 0,73–1,11) na categoria mais baixa de renda ( Tabela 2 ). A redução no consumo de arroz, pães, carnes processadas e refrigerantes foi mais acentuada na categoria de renda mais elevada, quando comparada à categoria de renda mais baixa. Por outro lado, a tendência de aumento do consumo de sanduíches foi maior na categoria de renda mais baixa (RC = 3,54; IC95% 2,83–4,42).

**Tabela 2:** Razão de chances ajustada a , b e intervalo de confiança de 95% de consumo de grupos alimentares segundo categorias de salários-mínimos per capita, Inquéritos Nacionais de Alimentação 2008–2009 e 2017–2018.

Grupos de Alimentos	Salários-Mínimos (SM)			
	< 0,5 SM	0,5 a 1 SM	1 a 2 SM	> 2 SM
	RC (IC95%)	RC (IC95%)	RC (IC95%)	RC (IC95%)
Arroz	0,61 (0,40–0,94)	0,67 (0,58–0,77)	0,31 (0,23 –0,41)	0,35 (0,30–0,40)
Feijão	0,24 (0,13–0,43)	0,39 (0,32–0,48)	0,29 (0,26–0,32)	0,42 (0,35–0,50)
Carne bovina	0,58 (0,43–0,78)	0,55 (0,51–0,61)	0,67 (0,64–0,70)	0,71 (0,63–0,80)
Frutas	0,44 (0,27–0,69)	0,58 (0,49–0,67)	0,65 (0,52–0,82)	0,68 (0,64–0,72)
Laticínios	0,40 (0,29–0,55)	0,38 (0,31–0,48)	0,51 (0,48–0,54)	0,46 (0,41–0,52)
Pães	0,82 (0,68–0,98)	0,45 (0,39–0,53)	0,40 (0,35–0,45)	0,26 (0,23–0,29)
Carnes processadas	1,08 (0,88–1,34)	0,63 (0,52–0,76)	0,56 (0,51–0,63)	0,39 (0,35–0,43)
Refrigerantes	0,90 (0,73–1,11)	0,62 (0,55–0,69)	0,68 (0,58–0,78)	0,40 (0,31–0,51)
Sanduíches	3,54 (2,83–4,42)	3,09 (2,42–3,94)	2,81 (2,20–3,58)	2,75 (2,27–3,32)

SM: salário-mínimo.

a Ajustada por idade e sexo.

b Ano de referência: 2008–2009.

A Figura 2 mostra o consumo médio em gramas por dia dos grupos de alimentos segundo as faixas de renda. Os resultados mostram similaridades com as frequências de consumo no geral: redução do consumo de arroz, feijão, carne bovina, pães, frutas, laticínios, carnes processadas e refrigerantes, e aumento no consumo de sanduíches. Para a primeira categoria de renda (até ½ salário-mínimo per capita), observa-se redução do consumo médio de feijão, frutas e laticínios entre os inquéritos, sendo essa de 41,8 g/dia, 22,4 g/dia e 18,8 g/dia, respectivamente. Entretanto, houve aumento no consumo médio de pães nessa mesma categoria de renda, de 42,3 g em 2008–2009 para 52,1 g em 2017–2018. Na categoria de renda mais alta (mais de dois salários-mínimos per capita), houve importante redução no consumo médio de refrigerantes (de 135,7 g/dia para 76,2 g/dia) e de carnes processadas (de 9,9 g/dia para 5,1 g/dia), enquanto o consumo médio de sanduíches nesta mesma categoria de renda aumentou 10 g no período (18,5 g/dia para 28,8 g/dia).

**Figura 2:** Consumo alimentar médio (g/dia) segundo categorias de salários mínimos (SM) per capita, INA 2008–2009 e 2017–2018, Brasil.

## DISCUSSÃO

Este estudo detalhado sobre a evolução do consumo alimentar nos últimos 10 anos demonstra que a alimentação do brasileiro ainda é caracterizada pelo consumo de alimentos tradicionais, como arroz e feijão, e pela frequência elevada de ingestão de alimentos ultraprocessados, como biscoitos e refrigerantes. No entanto, no período de 2008–2009 a 2017–2018, observou-se redução na frequência de consumo de arroz, feijão, frutas, carne bovina, pães, laticínios, carnes processadas e refrigerantes, mas aumento na ingestão de sanduíches. Essa tendência foi observada independentemente de sexo, idade e faixa de renda.

Apesar de alimentos tradicionais da alimentação brasileira permanecerem como os itens mais consumidos, a redução do consumo desses alimentos entre os dois inquéritos chama atenção, principalmente para o grupo feijão, importante marcador de alimentação saudável no país. O feijão, alimento espontaneamente presente no prato da população, faz parte da cultura alimentar do Brasil. Sabe-se que padrões alimentares baseados em alimentos tradicionais da dieta brasileira, in natura e minimamente processados, estão relacionados a menor ocorrência de obesidade e menos risco de DCNT^9^.

Nossos achados corroboram resultados de outros estudos de representatividade nacional. Dados da POF mostraram que a disponibilidade domiciliar média per capita anual de arroz e de feijão sofreu redução de 37% em 2002–2003 e de 52% em 2017–2018^2^. Dados do Sistema de Vigilância de Fatores de Risco para Doenças Crônicas Não Transmissíveis (Vigitel) também evidenciaram redução no consumo regular de feijão entre 2006 e 2019: uma variação média anual de -0,58 ponto percentual entre homens e -0,57 entre mulheres^10^.

Por outro lado, a redução no consumo de carnes processadas e refrigerantes foi um aspecto positivo, tendo em vista que esses são alimentos com elevado grau de processamento, nutricionalmente desbalanceados, que favorecem o consumo excessivo de energia^11^. Essa redução também foi observada pelo Vigitel entre 2006 e 2019, com variação média anual na frequência de consumo de refrigerantes de -1,52 ponto percentual entre homens e -1,30 entre mulheres^10^.

A redução no consumo tanto de alimentos tradicionais como de ultraprocessados pode ter sido impulsionada pela crise econômica iniciada em 2015^12^, que aumentou a insegurança alimentar no país. Segundo dados da POF 2017–2018, a prevalência de insegurança alimentar passou de 22,6% em 2013 para 36,7% em 2018, e a insegurança alimentar grave aumentou em 43,7% no período^5^.

O aumento na frequência de consumo de sanduíches também merece ser discutido, pois reflete uma importante mudança nos hábitos alimentares, caracterizada pela substituição de refeições tradicionais por lanches e alimentos de conveniência. Em muitas ocasiões, sanduíches são preparados com produtos ultraprocessados, que apresentam alto teor calórico e excesso de sódio ou açúcar, o que pode impactar negativamente a saúde^13^.

As mudanças observadas no consumo alimentar foram semelhantes entre as faixas etárias, mas a redução do consumo de frutas foi mais acentuada entre adolescentes. Diferenças geracionais no consumo de frutas e outros alimentos saudáveis já foram observadas anteriormente^14^. Idosos e adultos mais velhos são mais propensos a fazer mudanças positivas de dieta devido à maior compreensão dos benefícios da alimentação adequada ou à presença de doenças crônicas que exigem a adoção de hábitos mais saudáveis^15^.

A avaliação do consumo segundo as faixas etárias revela que a alimentação dos adolescentes já apresentava características negativas em comparação com a dos idosos no inquérito de 2008–2009. Após 10 anos, a alimentação dos adolescentes permanece de qualidade inferior à dos idosos, o que é coerente com o aumento da ocorrência de sobrepeso e obesidade entre adolescentes brasileiros nos últimos anos^16^. O excesso de peso na adolescência traz prejuízos importantes ao longo da vida, pois as características comportamentais e de estilo de vida adquiridas nessa fase tendem a perdurar na vida adulta^16^.

Outro dado preocupante a se destacar é o aumento mais acentuado na frequência de consumo de sanduíches entre idosos, tendo em vista os pontos já levantados (a substituição de refeições por lanches e o uso de produtos ultraprocessados como ingredientes). O consumo desses alimentos já era maior entre os adolescentes no primeiro inquérito. Logo, o aumento na frequência do consumo de sanduiches entre esse grupo foi menor em comparação com o aumento elevado entre adultos e idosos.

O período de 10 anos entre os inquéritos analisados também inclui mudanças na composição etária da população, o que pode ter influenciado achados relativos a mudanças nos hábitos alimentares. A expectativa de vida dos brasileiros aumentou mais de três anos nesse período, refletindo melhoria na qualidade de vida, o que favorece comportamentos menos limitantes entre idosos^17^. Isso também foi observado no aumento do consumo e contribuição calórica da alimentação fora de casa entre idosos, reforçando a hipótese de que a maior socialização os aproxima de comportamentos de adultos mais jovens^18^. Neste estudo, para a análise de tendências, os modelos foram ajustados por idade a fim de minimizar o efeito dessa variável nas mudanças de padrões alimentares. Além disso, apresentaram-se os efeitos das mudanças estratificados por faixa etária.

A variação da ingestão dos grupos de alimentos avaliados não foi uniforme nas diferentes faixas de renda. A diminuição no consumo de arroz, pães, carnes processadas e refrigerantes foi mais acentuada no grupo de renda mais elevada. Porém, foi observada tendência inversa, não estatisticamente significante, para feijão, carne bovina e frutas, cujas reduções mais acentuadas aconteceram na faixa de renda mais baixa.

Resultados similares foram observados nos dados de disponibilidade domiciliar no período entre 2008–2009 e 2017–2018 para o maior quinto de renda: enquanto a participação calórica do arroz diminuiu (de 12,3% para 10,9%), a contribuição calórica das frutas aumentou (de 3,1% para 3,9%)^2^. Esses dados confirmam as disparidades de hábitos entre níveis socioeconômicos, evidenciando a importância de políticas voltadas para a melhoria das condições de vida, como o Benefício de Prestação Continuada e o Programa Bolsa Família, que aumentam o acesso a alimentos saudáveis^19^. Também têm sido discutidas estratégias para limitar o acesso a alimentos não saudáveis, como refrigerantes^20 , 21^.

Algumas limitações deste estudo precisam ser consideradas. Mudanças na base de dados, como a inserção de diversos itens no segundo inquérito, podem ter influenciado os achados – por exemplo, no aumento observado de consumo de sanduíches. No INA 2008-2009, havia 17 códigos disponíveis para captar o consumo de sanduíches, enquanto em 2017–2018 esse número passou para 61. Outra possível limitação está relacionada aos diferentes métodos aplicados para coletar as informações de consumo alimentar: em 2008–2009, utilizou–se o registro alimentar, e em 2017–2018 o recordatório alimentar de 24 horas. Rodrigues et al. porém, mostraram que é possível analisar a evolução do consumo independentemente das metodologias usadas em cada inquérito, usando estratégias de análises para harmonização^22^.

Um ponto forte do estudo é a estimativa do consumo alimentar usual dos indivíduos. Os dois dias de consumo coletados nos dois inquéritos permitiram estimar a variabilidade intraindividual, incorporando nas análises de tendência o método do NCI, que permite estimar a distribuição e os efeitos de covariáveis não dietéticas no consumo usual dos alimentos.

O trabalho, que investigou o consumo alimentar efetivo de indivíduos acima de 10 anos de idade, é o primeiro a avaliar tendências de consumo alimentar em amostra representativa da população brasileira com base em dados individuais de consumo alimentar global. Devido ao detalhamento de informações sobre preparações e adições específicas a alimentos consumidos individualmente, as análises apresentadas superam o viés de tendências estimadas a partir de dados de disponibilidade de alimentos.

As mudanças na última década, caracterizadas pela redução do consumo de alimentos tradicionais, como arroz, feijão e carne, e aumento do consumo de sanduíches, sinalizam piora na alimentação do brasileiro. Apesar da redução no consumo de alguns alimentos ultraprocessados, como refrigerantes e biscoitos, esses itens ainda estão entre os alimentos mais consumidos no país. Tais resultados indicam que são necessárias medidas econômicas e de saúde pública que permitam e estimulem o consumo de feijão, frutas e hortaliças (importantes marcadores de qualidade da alimentação), ao mesmo tempo em que desencorajem o consumo de ultraprocessados. Essas medidas aumentariam a segurança alimentar e nutricional e preveniriam as DCNT, entre elas a obesidade.

## Footnotes

a Instituto Brasileiro de Geografia e Estatística, Diretoria de Pesquisas, Coordenação de Trabalho e Rendimento. Pesquisa de Orçamentos Familiares, 2017–2018: análise do consumo alimentar pessoal. Rio de Janeiro: IBGE; 2020 [citado 28 out 2020]. Disponível em: https://biblioteca.ibge.gov.br/visualizacao/livros/liv101742.pdf

b National Institutes of Health (US), National Cancer Institute, Division of Cancer Control & Population Sciences. Epidemiology and Genomics Research Program. Usual dietary intakes: SAS macros for analysis of a single dietary compoment. Bethesda, MD: NCI; 2020 [citado 14 set 2020]. Disponível em: https://epi.grants.cancer.gov/diet/usualintakes/macros_single.html
